# Hysteresis of Low-Temperature Thermal Conductivity and Boson Peak in Glassy (g) As_2_S_3_: Nanocluster Contribution

**DOI:** 10.1186/s11671-017-2125-6

**Published:** 2017-05-10

**Authors:** V. Mitsa, A. Feher, S. Petretskyi, R. Holomb, V. Tkac, P. Ihnatolia, A. Laver

**Affiliations:** 1 Uzhhorod National University, Pidhirna Str., 46, Uzhhorod, 88000 Ukraine; 2 Pavol Jozef Šafárik University in Košice, 041 54 Košice, Slovak Republic; 3 Department of Condensed Matter Physics, Faculty of Mathematics and Physics, Charles University, Ke Karlovu 5, CZ-12116 Prague 2, Czech Republic

**Keywords:** Chalcogenide glass, Thermal conductivity, Low temperatures, Nanostructured semiconductors, Boson peak, Raman spectroscopy, 61.43.Fs, 61.43.Bn, 31.15.A-, 63.50.Lm, 44.10. + i, 65.60. + a

## Abstract

Experimental results of the thermal conductivity (*k*(*T*)) of nanostructured g-As_2_S_3_ during cooling and heating processes within the temperature range from 2.5 to 100 K have been analysed. The paper has considered thermal conductivity is weakly temperature *k*(*T*) dependent from 2.5 to 100 K showing a plateau in region from 3.6 to 10.7 K during both cooling and heating regimes. This paper is the first attempt to consider the *k*(*T*) hysteresis above plateau while heating in the range of temperature from 11 to 60 K. The results obtained have not been reported yet in the scientific literature. Differential curve Δ*k*(*T*) of *k*(*T*) (heating *k*(*T*) curve minus cooling *k*(*T*) curve) possesses a complex asymmetric peak in the energy range from 1 to 10 meV. Δ*k*(*T*) reproduces the density of states in a *g*(*ω*)/*ω* ^2^ representation estimated from a boson peak experimentally obtained by Raman measurement within the range of low and room temperatures. Theoretical and experimental spectroscopic studies have confirmed a glassy structure of g-As_2_S_3_ in cluster approximation. The origin of the low-frequency excitations resulted from a rich variety of vibrational properties. The nanocluster vibrations can be created by disorder on atomic scale.

## Background

Chalcogenide glass (g) g-As_2_S_3_ is a canonical infrared optical material for practical applications in chalcogenide photonics. Thermal conductivity (*k*) is a property determining the working temperature levels of optical media during high-power infrared (IR) laser illumination [1]. Experimental discoveries of the low-temperature thermal anomalies of glasses have been first described in oxide glasses [2]. A few years later, anomaly at *T* < 1 K that was fixed with almost quadratic temperature dependence of thermal conductivity *k* was confirmed for g-As_2_S_3_ [3]. The anomaly at intermediate temperatures that range up to 10 K for g-As_2_S_3_ was discovered later [4]. Within this temperature range, the thermal conductivity exhibits a ubiquitous plateau [2, 3]. The glassy materials deviate from well-known universal thermal properties at low temperatures for crystalline solids [2]. The experimental findings of thermal conductivity measurements on glasses below 1 K have been interpreted by most successful model [5, 6] in the terms of “two-level” or “tunnelling” systems, which were later named as “standard tunnelling model” (STM) [7, 8]. Within the frame of STM model, it has been assumed that the “plateau” behaviour arises from the existence of quasi-localized low-frequency (LF) modes [9]. Many models have been proposed for further description of the structural origin of the LF modes [10–22]. A number of them are related to the nanoheterogeneous nature of glasses. Nanoheterogeneities generate an intermediate range ordering in the chalcogenide glasses. This relates to an excess in vibrational density of states’ contribution which is directly connected with the so-called boson peak (BP) observed by neutron or vibrational Raman spectroscopy [10, 11, 14, 18, 20, 21]. The theoretical explanation of the temperature dependence of the thermal conductivity coefficient for glasses in the temperature range above the plateau has been considered on the basis of intermediate range ordering [18]. Theoretical and experimental spectroscopic studies confirm a glassy structure of g-As_2_S_3_ in cluster approximation. The origin of the low-frequency excitations resulted from a rich variety of vibrational properties of nanocluster vibrations [20, 21, 23, 24].

The aim of this work is to analyse the dependencies of low-frequency modes from system cluster size. Our efforts have been focused on the LF vibrational modes which may be involved in the measured BP and thermal conductivity in g-As_2_S_3_. The experiment has been carried out to study thermal conductivity behaviour during cooling and heating within the temperature range from 2.5 to 100 K.

## Methods

The sample of optical quality was prepared by melting additionally purified elements of arsenic and sulphur in clean evacuated and sealed quartz ampoules placed at a rocking furnace at 600 °C for a period of 24 h. Then, it was cooled in the air with a cooling rate of 1 K/s. Such conditions of preparation minimized nanosize realgar cluster separation [25].

Thermal conductivity of the nanostructured g-As_2_S_3_ was studied between 2.5 and 100 K, during both cooling and heating procedures. The measurement was performed in commercial Quantum Design Physical Property Measurement System (PPMS) with a thermal transport option (TTO). Two-probe lead configuration has been chosen, due to the fact that the sample has small thermal conductivity. Pill-shaped sample (2.5 × 5 × 5 mm^3^) has been cut for these measurements. It was glued between two disk-shaped copper leads (hot and cold platforms) with GE Varnish glue. The heater and hot thermometer were attached against each other at the hot platform. The cold thermometer and thermal reservoir were mounted on cold platform in the same way as hot platform. Thermal conductivity of the sample is much smaller than the thermal conductivity of the leads. The heat power was applied from the heater mounted on the hot platform in order to create a user-specified temperature gradient between the two thermometers mounted on cold and hot platforms. For measuring the continuous measurement mode was selected. During cooling and heating procedures, the measurements are being taken continually and the adaptive software is adjusting the parameters (such as heater power) to optimize the measurements. In the first study cycle, *k*(*T*) 100 to 2.5 K temperature was held at cooling the sample with rate 0.385 K/min. The second series of measurements included both cooling with the rate of 0.385 K/min from 100 to 2.5 K temperature and heating with the rate of 0.415 K/min in the opposite direction. Based on thermal transport hardware and software, it was possible to calculate thermal conductivity directly from the applied heater power, resulting ∆*T*, and sample geometry using the equation:

1 $$ k=\frac{Pl}{S\left({T}_2-{T}_1\right)} $$

where *k* is the thermal conductivity (W/Km), *P* is applied heater power (W), *l* is the height of measured sample (m) and *S* is the cross sectional area of sample (m^2^).

**Fig. 1:**
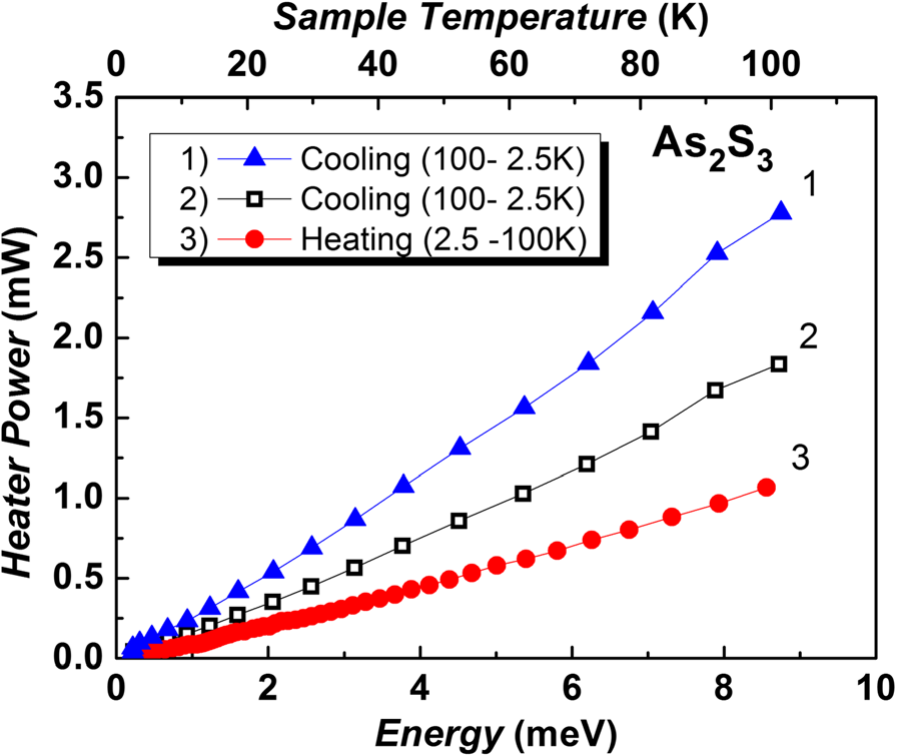
The heater power changes during the measurement processes (heating and cooling of g-As_2_S_3_) (see details in the *inset*). (*Color online*)

**Fig. 2:**
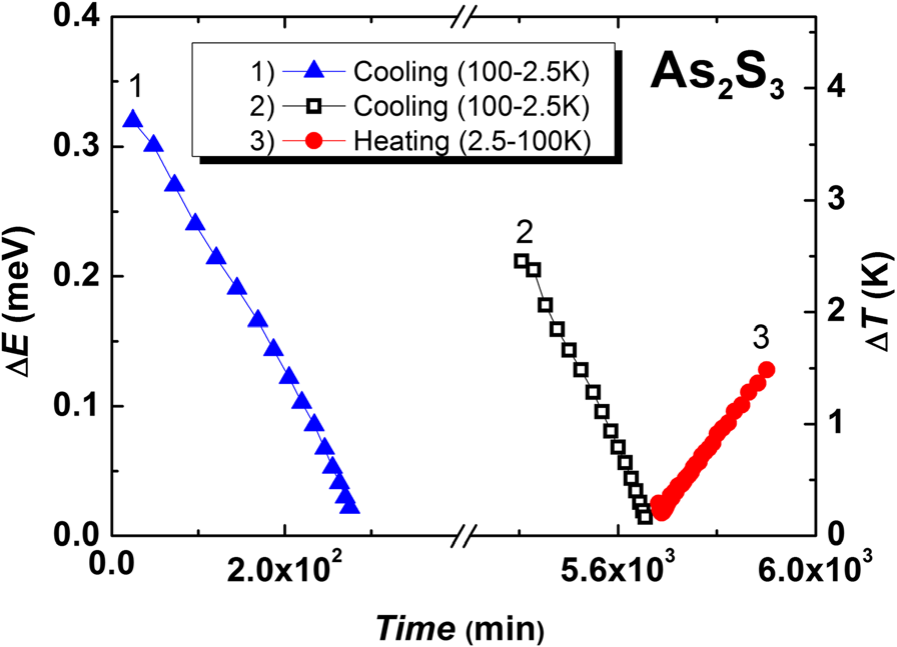
The time dependence of the temperature difference between the cold and hot ends of the sample in the process of measurement during cooling (*curves 1*, *2*) and heating (*curve 3*). (*Color online*)

The results were obtained when experimental conditions have been selected to obtain maximum temperature uniformity across the sample. Depending on the time, the heater temperature and resulting ∆*T* have demonstrated linear trend for both series of measurements (Figs. 1 and 2). The accuracy of the measurement is equivalent to 3%. This paper focuses on the results taken from 2.5 to 100 K.

Room-temperature low-frequency Raman spectra were measured using a triple grating Dilor-XY800 spectrometer equipped with a CCD detector cooled by liquid nitrogen. The slit width was set to 1 cm^−1^. Laser line of 632.8 nm was used as the excitation source.

**Fig. 3:**
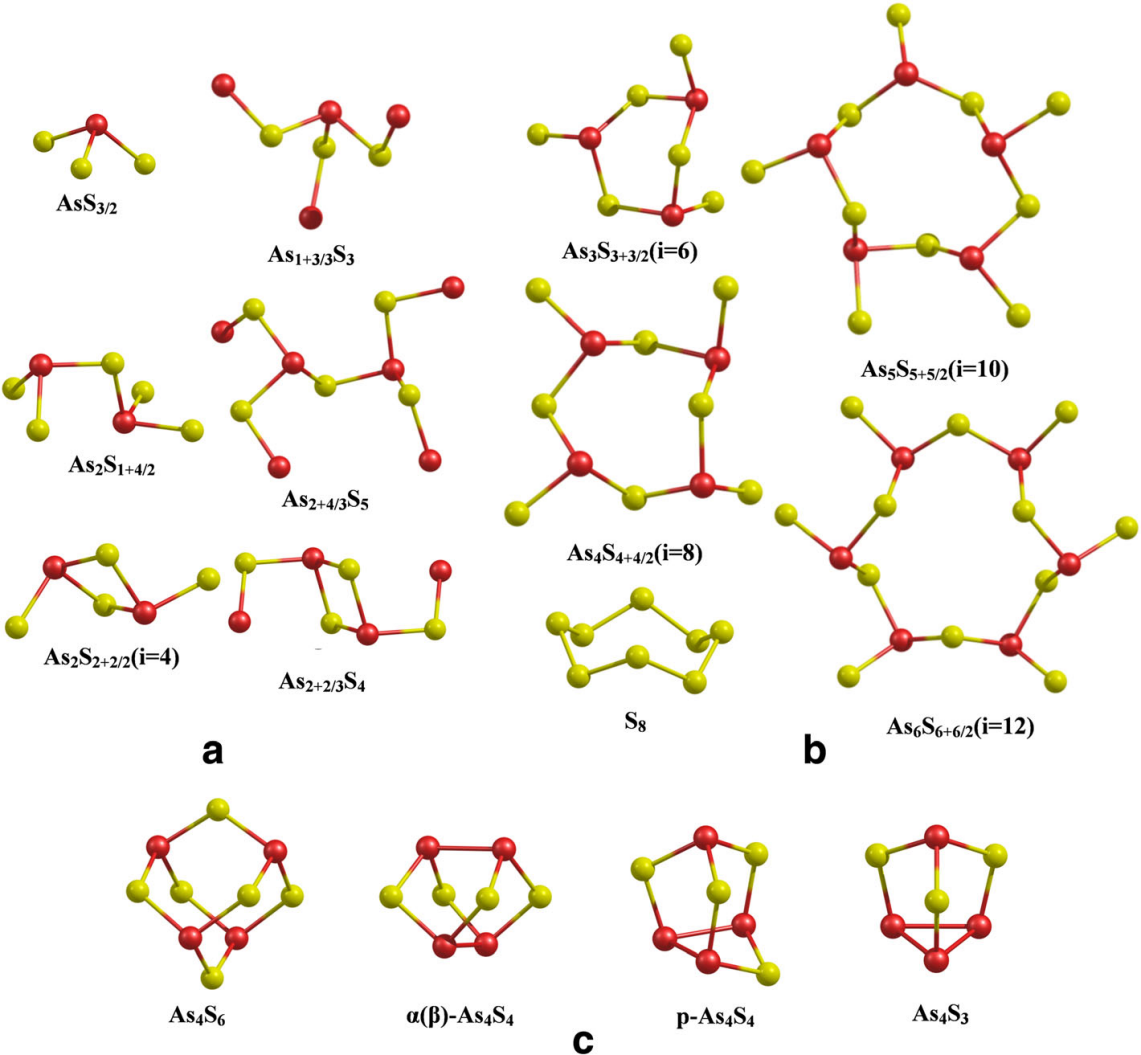
Branchy- (**a**), ring- (**b**) and cage-like (**c**) As_n_S_m_ nanocluster models. Saturating hydrogen atoms for branchy- and ring-like nanoclusters are not shown for clarity. (*Color online*)

Finite size atomic As_n_S_m_ nanoclusters containing structural units are expected to be important for glassy As-S system. For better modelling of the chemical environment, the dangling bonds of clusters were terminated by H atoms. These assumptions were used for the Raman study of active LF modes (Fig. 3). Density functional theory (DFT) calculations of optimal geometry, total and formation energies and electronic and vibrational properties of these clusters were performed, using GAMESS (US) program [26]. The pure corrected exchange functional proposed by Becke (B) [27] and the gradient-corrected correlation functional proposed by Lee, Yang and Parr (LYP) [28] were applied for calculations. The modified Stuttgart RLC ECP basis set [29] was used for As and S atoms. The contribution of terminal H atoms’ vibrations in the calculated Raman spectra of As_n_S_m_ nanoclusters were subsequently eliminated. The details of basis set modification and spectral treatment have been already described [20, 21, 24].

## Results and Discussion

**Fig. 4:**
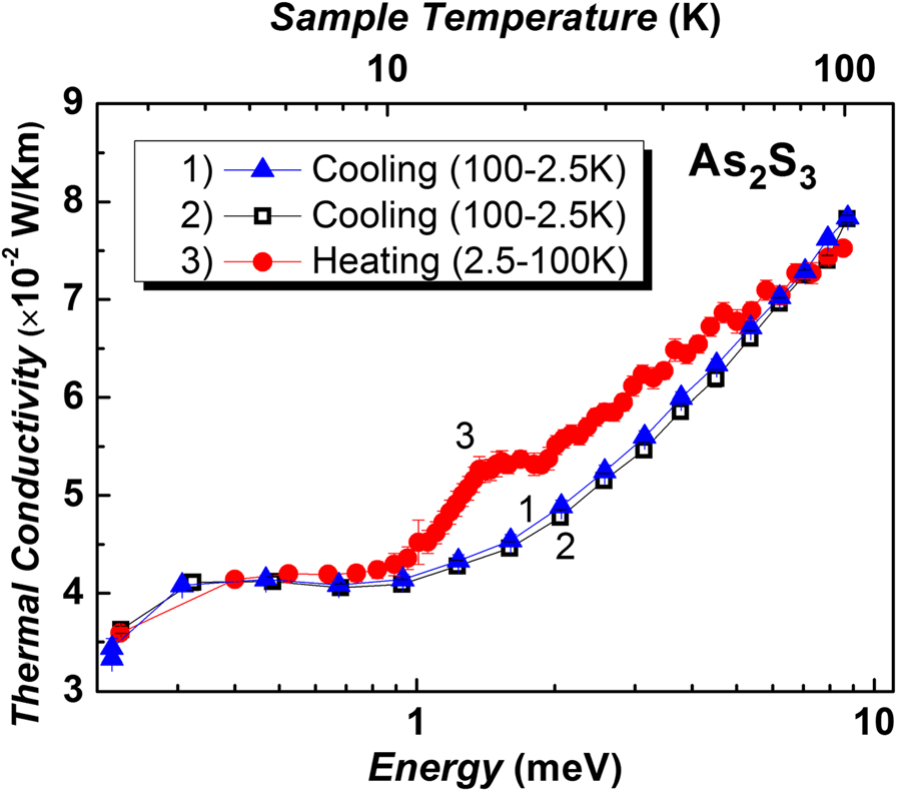
Low-temperature thermal conductivity of g-As_2_S_3_ during cooling (*curve 1* and *2*) and heating (*curve 3*) cycles. (*Color online*)

**Fig. 5:**
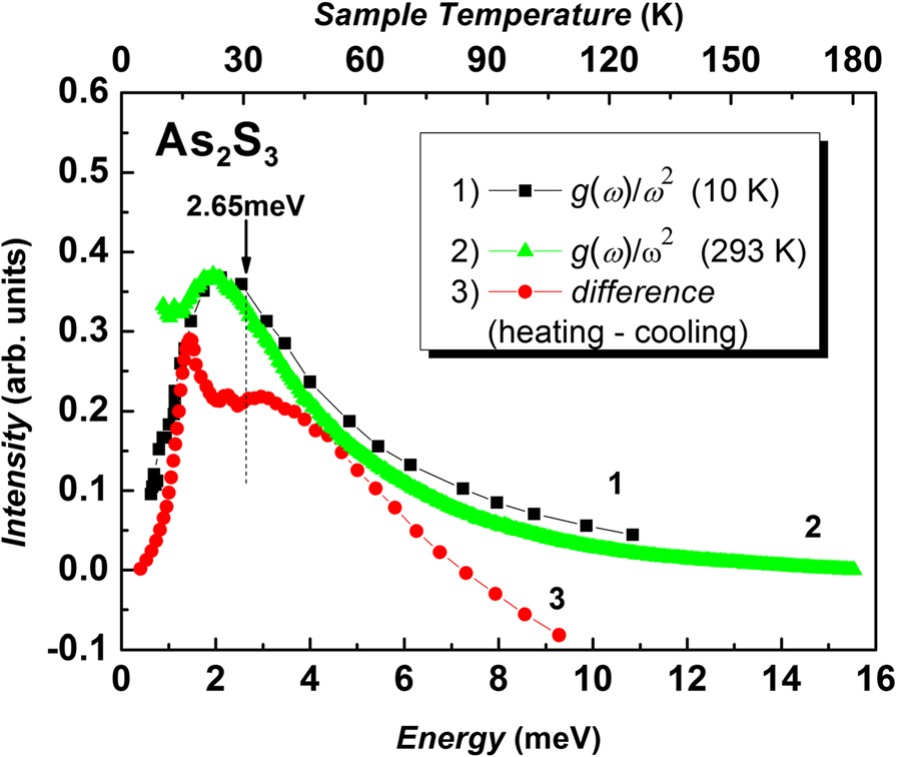
Density of states *g*(*ω*) in representation *g*(*ω*)/*ω* ^2^ estimated from measured low-frequency (LF) Raman spectra at 10 K taken from [11] (*curve 1*); *g*(*ω*)/*ω* ^2^ from own LF Raman spectra measured at room temperature (293 K) (*curve 2*) and difference curve *k*(*T*) between cooling *k*(*T*) and heating *k*(*T*) cycles (see Fig. 4) (*curve 3*). (*Color online*)

The structural origin of low-temperature thermal conductivity and boson peak are still open for discussion and are the matter of debate [10–22]. The thermal conductivity in g-As_2_S_3_ studied between 2.5 and 100 K during cooling and heating procedure is shown at Fig. 4. The measured values of thermal conductivity agree with the data for chalcogenide glasses described earlier [15]. As it can be seen, the thermal conductivity *k*(*T*) is slightly temperature dependent from 2.5 to 10 K showing a plateau region during both cooling and heating regimes (see Fig. 4). For temperature range mentioned above, the similar plateau in g-As_2_S_3_ has been found before [4]. The linear relationship with a tg(*α*) = 0.0003 slope was found for the temperature dependencies *k*(*T*) above the plateau (11–100 K) independently on different cooling cycles within the accuracy of measurement. At the same time, the jump in *k*(T) dependence between cooling and heating cycles of g-As_2_S_3_ has been discovered (see Fig. 4). The values show that the jump of *k*(*T*) during heating is greater than the accuracy of the measurement producing an appreciable deviation from *k*(*T*) values taken during the cooling. The appearance of hysteresis in *k*(*T*) during heating was fixed in the temperature range from 11 to 60 K. The difference curve Δ*k*(*T*) of *k*(*T*) (heating minus cooling) has a complex asymmetric peak when energy is between 1 and 10 meV (Fig. 5) and reproduces the experimental low-temperature boson peak (excess of the density of states based on the Debye prediction). The BP seen by Raman measurements is shown in Fig. 5 in scale *g*(*ω*)/*ω* ^2^ (curve 1, *T* = 10 K [11], curve 2, *T* = 293 K). The position of *g*(*ω*)/*ω* ^2^ maximum at 2.65 eV in the experimental neutron scattering measurements of g-As_2_S_3_ density of state *g*(*ω*) is indicated in Fig. 5 by arrow for the comparison. However, in addition to complex experimental investigations of *g*(*ω*) by neutron scattering [10], the position of maximum *g*(*ω*)/*ω* ^2^ mentioned above can be estimated by measuring the LF Raman spectra of glasses at 10 K (Fig. 5, curve 1) when contribution of quasi-elastic light scattering to the Raman spectra at below 10 cm^−1^ is negligible. According to theoretical calculations, the experimentally observed intensity (*I*_exp_) in the low-frequency spectrum is given by [11]:

2 $$ {I}_{\exp}\left(\varDelta \omega \right)=\frac{C\left(\varDelta \omega \right) g\left(\varDelta \omega \right)\left[ n\left(\varDelta \omega \right)+1\right]}{\varDelta \omega}, $$

where *C*(∆*ω*) is light-to-vibrations coupling coefficient, ∆*ω* is a Raman shift and *g*(∆*ω*) is the density of state; $n\left(\varDelta \omega \right)=\frac{1}{{ \exp}^{\left(\frac{h\varDelta \omega}{kT}\right)}-1}$ − Bose factor for the Stokes component.

Taking into account the mentioned above, the reduced intensity

3 $$ {I}_{\mathrm{red}}\left(\varDelta \omega \right)=\frac{I_{\exp}\left(\varDelta \omega \right)}{\varDelta \omega \left[ n\left(\varDelta \omega \right)+1\right]}=\frac{C\left(\varDelta \omega \right) g\left(\varDelta \omega \right)}{{\left(\varDelta \omega \right)}^2}. $$

Based on neutron scattering measurements [10], the coupling coefficient *C*(∆*ω*) between the reduced Raman intensity (*I*_red_) and the *g*(*∆ω*) is believed to be a monotonically increasing function *C*(∆ω) ≈ ∆ω from 5–8 up to 100 cm^−1^. In this case, the boson peak seen by Raman measurements is due to an anomaly in *g*(∆*ω*) and reflecting an enhancement of low-frequency states relative to an elastic continuum Debye level [11].

It is known that the BP in the low-frequency Raman spectra of glasses has also been related to the existence of intermediate range ordering (clusters) [18, 23, 24]. To understand how these low-frequency modes depend on the type and system size, several As-S clusters (namely branchy-, ring- and cage-like) were modelled (Fig. 3) and used to calculate the LF Raman active modes. It was found that among the clusters presented in Fig. 3, only the glass-network forming branchy- (a) and ring-like (b) clusters exhibit LF vibrational modes. No LF vibrational modes were found in the calculated Raman spectra of the rigid cage-like As-S nanoclusters (Fig. 3c).

**Fig. 6:**
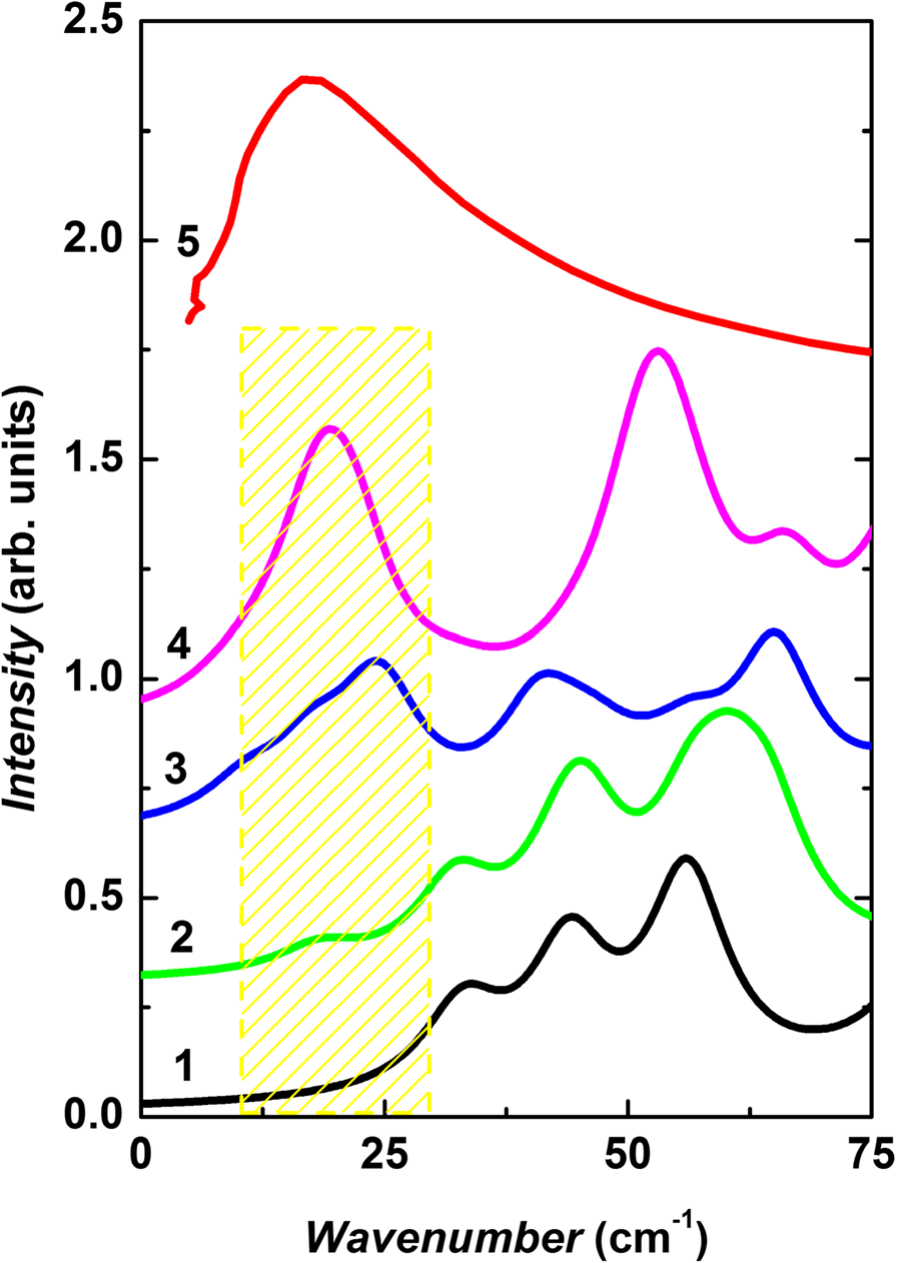
Simulated low-frequency Raman spectra of As_n_S_m_ ring-like clusters (*1*–*4*): (*1*) As_6_S_6+6/2_, (*2*) As_5_S_5+5/2_, (*3*) As_4_S_4+4/2_, (*4*) As_3_S_3+3/2_ and *g*(*ω*)/*ω* ^2^ (10 K, ref. [11]) of g-As_2_S_3_ (*5*). (*Color online*)

Results have shown that a single pyramidal structural unit can be characterized by stretching and deformation type vibrations only, i.e*.* no LF modes were calculated for AsS_3/2_ cluster. However, the further branching of this cluster leads to the appearance of LF vibrational modes in the calculated Raman spectra of As_1+3/3_S_3_ cluster at 23.5, 39.3 and 46.8 cm^−1^. A correlation between LF modes and cluster size was also found when branching of As_2_S_1+4/2_ cluster takes place (see Fig. 3). For As_2_S_1+4/2_ cluster, LF Raman active modes were calculated at 31.7, 43.1 and 58.8 cm^−1^. The number of LF vibrations of the branched structure (cluster As_2+4/3_S_5_) increases, and their frequencies shift towards low-frequency region: 12.8, 17.3, 23.5, 30.2, 32.8, 36.3 and 40.1 cm^−1^. Similar situation can be found when calculated LF Raman active modes of As_2_S_2+2/2_ and As_2+2/3_S_4_ are compared. The LF Raman spectra simulated with Lorenz curves (full width at half maximum is 10 cm^−1^) calculated for ring-like As-S nanoclusters are shown in Fig. 6. Both the frequency position and Raman intensity of the calculated bands of As-S rings show strong dependence on cluster size (i). As it can be seen, the most intensive band in the calculated LF Raman spectra of As_3_S_3+3/2_ cluster is located at ~56.1 cm^−1^. With the increasing cluster size, the new Raman bands start to appear at lower frequency region of the simulated spectra (see Fig. 6). Two LF Raman bands centred at 19.7 and 53.4 cm^−1^ can clearly be seen in the simulated Raman spectra of 12-membered As-S ring (cluster As_6_S_6+6/2_).

The lowest vibrational mode of 6-, 8-, 10- and 12-membered rings (Fig. 3) is less intensive in the Raman spectra and is located at 33.1, 18.2, 10.8 and 9.0 cm^−1^, respectively. The analysis of normal coordinates of these low-frequency vibrations indicate that they are torsional and out of plane bending vibrations involving group of atoms (within 3–5 bonds). The atomic motions for some of these vibrations have a “wavelike” character. The larger *i*-member rings and branchy As_n_S_m_ nanoclusters can be associated with the intermediate range order of As_2_S_3_ glass and produce the localized collective LF vibrations. This can be responsible for the low-temperature anomalies and BP of g-As_2_S_3_. Quasi-localized modes that resonantly couple with transverse phonons might lead to the accumulation of the low-energy modes around the boson peak [22].

It is important to note that with increasing the number of atoms in branched clusters (As_2_S_3_)*n*, *n* = 1−3, the energy formation decreases from −41.5 (cluster As_2_S_3_) to −124.6 Hartree (cluster As_6_S_9_). Lowering the formation energy of large clusters demonstrates the higher probability of such structure formation [30]. Calculated low-frequency vibrations of As_n_S_m_ clusters demonstrate that the collective torsional and out of plane bending vibrations of the big 10- and 12-membered rings (10.8 and 9.0 cm^−1^, respectively) and torsional vibrations of As_2+4/3_S_5_ branched A_n_S_m_ clusters (12.8 cm^−1^) can contribute to *g(ω)/ω* ^*2*^ (Fig. 6) and *k*(*T*) above the plateau in g-As_2_S_3_ (Fig. 4).

## Conclusions

The temperature dependence of the thermal conductivity *k*(*T*) of g-As_2_S_3_ has been studied within the temperature range from 2.5 to 100 K during cooling and heating cycles. The presence of “plateau” in the *k*(*T*) ranged from 3.6 to 10.7 K (0.31–0.92 meV) has been confirmed. Within the temperature range from 11 to 60 K, the hysteresis of temperature dependence during cooling and heating procedure has been observed above plateau. Dependencies *k*(*T*) for different cooling cycles in the temperature range from 11 to 100 K, which is located above the plateau, within the accuracy of measurement have linear behaviour with a slope tg(*α*) = 0.0003. The contribution to thermal conductivity and boson peak in the Raman spectra can have torsion and out of plane bending vibrations of ring and branched clusters’ origin.

## Abbreviations

BP: Boson peak
LF: Low frequency
STM: Standard tunnelling model
